# Probing excitonic states in suspended two-dimensional semiconductors by photocurrent spectroscopy

**DOI:** 10.1038/srep06608

**Published:** 2014-10-16

**Authors:** A. R. Klots, A. K. M. Newaz, Bin Wang, D. Prasai, H. Krzyzanowska, Junhao Lin, D. Caudel, N. J. Ghimire, J. Yan, B. L. Ivanov, K. A. Velizhanin, A. Burger, D. G. Mandrus, N. H. Tolk, S. T. Pantelides, K. I. Bolotin

**Affiliations:** 1 Department of Physics and Astronomy, Vanderbilt University, Nashville, TN-37235, USA; 2 Interdisciplinary Graduate Program in Materials Science, Vanderbilt University, Nashville, TN-37234, USA; 3 Department of Physics and Astronomy, University of Tennessee, Knoxville, TN-37996, USA; 4 Materials Science and Technology Division, Oak Ridge National Laboratory, Oak Ridge, TN-37831, USA; 5 Department of Materials Science and Engineering, University of Tennessee, Knoxville, TN-37996, USA; 6 Theoretical Division, Los Alamos National Laboratory, Los Alamos, NM-87545, USA; 7 Department of Physics, Fisk University, Nashville, TN-37208, USA

## Abstract

The optical response of semiconducting monolayer transition-metal dichalcogenides (TMDCs) is dominated by strongly bound excitons that are stable even at room temperature. However, substrate-related effects such as screening and disorder in currently available specimens mask many anticipated physical phenomena and limit device applications of TMDCs. Here, we demonstrate that that these undesirable effects are strongly suppressed in suspended devices. Extremely robust (photogain > 1,000) and fast (response time < 1 ms) photoresponse allow us to study, for the first time, the formation, binding energies, and dissociation mechanisms of excitons in TMDCs through photocurrent spectroscopy. By analyzing the spectral positions of peaks in the photocurrent and by comparing them with first-principles calculations, we obtain binding energies, band gaps and spin-orbit splitting in monolayer TMDCs. For monolayer MoS_2_, in particular, we obtain an extremely large binding energy for band-edge excitons, *E*_bind_ ≥ 570 meV. Along with band-edge excitons, we observe excitons associated with a van Hove singularity of rather unique nature. The analysis of the source-drain voltage dependence of photocurrent spectra reveals exciton dissociation and photoconversion mechanisms in TMDCs.

Monolayer (1L) transition metal dichalcogenides (TMDCs), such as molybdenum disulfide (MoS_2_), molybdenum diselenide (MoSe_2_), or tungsten diselenide (WSe_2_) are two-dimensional atomic crystals^1^,^2^,^3^. In contrast to graphene^4^, a prototypical 2D material, 1L-TMDCs are direct band gap semiconductors with strong spin-orbit interactions, which cause spin-splitting of the valence band of TMDCs^5^,^6^,^7^ and allow optical manipulation of spin- and valley- degrees of freedom in these materials^8^,^9^,^10^,^11^. Two-dimensional confinement, high effective carrier mass and weak screening lead to strong electron-electron interactions and dominance of tightly bound excitons in the optical properties of 1L-TMDCs^5^,^6^,^7^,^8^,^9^,^10^,^11^,^12^,^13^,^14^. These extraordinary properties make TMDCs ideal platform for studying many anticipated phenomena including quantum-, valley- and spin-Hall effects^6^,^15^,^16^, superconductivity in monolayer MoS_2_^17^,^18^ and many-body effects^12^,^13^,^19^. Moreover, strong light-matter interactions^20^ make TMDCs excellent materials for ultrasensitive photodetectors^21^,^22^,^23^,^24^ and energy harvesting devices^25^. Despite rapid progress in understanding the electronic and optical properties of TMDCs^1^, important fundamental questions remain unanswered:

1. What types of excitons exist in TMDCs and what are their binding energies? While calculations predict a plethora of excitonic states with extremely large binding energies^26^,^27^, experimental progress has been hampered by large broadening of the excitonic peaks in the available samples^5^,^26^.
2. How do substrate-related effects perturb the intrinsic properties of monolayer TMDCs? Indeed, there are indications that the presence of a substrate can cause strong carrier scattering^28^,^29^ and affect exciton energies through screening^30^.
3. What are the photoconversion mechanisms in TMDC devices? Despite indications of efficient photoconversion^20^,^21^, photodetection^21^,^22^,^23^,^24^, and strong interest in employing TMDCs as solar cells^25^, it is currently unclear how strongly-bound excitons in TMDCs dissociate and contribute to the photocurrent.

Our experiments are geared towards answering these questions. First, we eliminate substrate-related screening in TMDCs by fabricating free-standing and electrically contacted MoS_2_, MoSe_2_, and WSe_2_ specimens. We then use photocurrent spectroscopy as a versatile tool for studying excitons and their dissociation mechanisms. In monolayer (1L) MoS_2_, we have observed well-defined peaks at ~1.9 eV and ~2.1 eV (‘A’ and ‘B’) and a broad peak ‘C’ at ~2.9 eV. We attribute the peaks A and B to optical absorption by band-edge excitons, and the peak C to absorption by excitons associated with the van Hove singularity of MoS_2_. Compared to previously reported optical absorption measurements of supported MoS_2_^5^, our photocurrent spectra exhibit sharp and isolated peaks with near-zero background between them, suggesting the absence of disorder-related midgap states. Our suspended devices allow us to obtain experimentally, for the first time, the lower bound of the binding energy of band-edge excitons of MoS_2_, *E*_bind_ ≥ 570 meV. Finally, we investigate the photoconversion and photogain mechanisms in monolayer TMDCs. By controlling the source-drain voltage, we observe different dissociation pathways for A/B- and C-excitonic states, demonstrate photogain of the order of 1000 with response times faster than 1 ms, and investigate the mechanism of this photogain. We also demonstrate the universality of our techniques by performing measurements on other materials, such as bi- and multi-layer MoS_2_, monolayer MoSe_2_ and monolayer WSe_2_. Our results demonstrate, for the first time, that photocurrent spectroscopy is an efficient tool for probing single- and many-body states in pristine TMDCs and suggest the application of TMDCs as efficient photodetectors with a voltage-tunable spectral response.

In attempt to decrease the substrate-induced screening and disorder in TMDCs, we studied 14 electrically contacted suspended devices with typical dimensions ~1 μm × 1 μm made from different TMDCs following the approach developed for graphene^28^ (see Supplementary Information, S1 for details). Initially, we focus on 1L-MoS_2_ devices (Fig. 1a, Inset), while discussing the case of monolayer MoSe_2_, WSe_2_, and multilayer MoS_2_ later. Two-probe electrical transport measurements indicate that upon suspension the field effect carrier mobility (*μ*) of a typical device (device #1), ~0.05 cm^2^/Vs, increases by an order of magnitude (Fig. 1a), consistent with a recent report^29^. We note that since neither the contact resistance nor the carrier density can be determined in the two-probe geometry, the physically relevant Hall or four-probe mobility of the same device may be larger by orders of magnitude^31^,^32^,^33^. To further increase the quality of suspended devices, we rely on thermal annealing, which is effective in improving *μ* both for graphene^28^ and multilayer MoS_2_^34^. Since the low electrical conductance (*G*) of MoS_2_ devices precludes annealing via Ohmic heating^28^, we instead locally heat the region of the wafer that is in thermal contact with the device. The annealing is performed *in situ* inside a cryostat kept at base temperature *T = 77 K* using a ~5 W CO_2_ laser beam, which is defocused (intensity < 20 μW/μm^2^) to avoid sample damage. Annealed and unannealed MoS_2_ samples were imaged with atomic resolution using aberration-corrected scanning transmission electron microscopy. We did not observe any annealing-induced modification or defects (see Supplementary Information, S2). This annealing renders the device near-insulating under small source-drain bias voltage *|V_ds_| < 1 V* (Fig. 1a, red curve). This behavior is consistent with a pristine undoped semiconductor with the Fermi level located inside the band gap. Since the gate voltage is limited to |*V_g_*| < *12 V* to avoid electrostatic collapse of MoS_2_, we are unable to achieve either electron or hole conductivity regimes via electrostatic gating.

To investigate suspended devices further, we measure PC under high *V_ds_ (>3* V*)* (Fig. 1b, blue curve). We illuminate the entire device using a low intensity (≤30 pW/μm^2^) light source and record photocurrent *I_PC_* across the device as a function of the photon energy 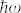 (Fig. 1c). The total current through the device is *I = V_ds_ G(V_ds_, n)*, where *G* in turn depends on the number of charge carriers *n* and *V_ds_*. Upon illumination with power *P*, *n* increases by 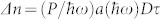, where *α* is the absorption coefficient, *D* is the photoconversion probability (the probability of generating an unbound photocarrier by an absorbed photon), and *τ* is the photocarrier lifetime^35^. For a constant *V_ds_*, the photocurrent is 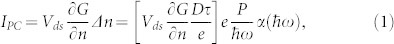
where *e* is the electron charge. The expression inside the brackets is the photogain *η*, the ratio between the number of photocarriers transported across the device and the number of absorbed photons per unit time. Assuming *α*(1.9 eV) ~ 0.1 and *α*(2.9 eV) ~ 0.4^5^ we estimate *η* ~ 200 at *V_ds_* ~ 10 V, for a device #2 (Figs. 1b). In another device #4 we observed *η* > 1,000.

Equation (1) is central to the analysis of our data as it shows that PC can be used to estimate the intrinsic parameters of TMDCs 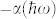, *τ*, and *D*. Indeed, since the photogain is weakly wavelength-dependent, peaks in *I_PC_* are associated with peaks in 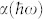 (See the Supplementary Information, S4 for more detail). On the other hand, the amplitude of *I_PC_* is related to photogain, and hence to *D* and *τ*. Therefore, similarly to optical absorption measurements, PC spectroscopy allows us to study single- and many-body electronic states in TMDCs^36^,^37^. Unlike absorption spectroscopy, PC can be easily measured for an electrically contacted microscopic device in a cryogenic environment, as the device itself acts as its own photodetector. Moreover, high photosensitivity of TMDC phototransistors allows us to use very low illumination intensity in our experiments, thereby excluding artifacts, such as photo-thermoelectric effects^38^ (which would yield currents <0.1 pA, more than three order of magnitude smaller than the photocurrent measured in our devices) and optically non-linear^39^ effects arising at high photocarrier densities. We first use PC spectroscopy to probe absorption spectrum 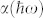 of TMDCs, while later investigating the origins of large photogain.

For substrate-supported and for majority of suspended unannealed devices, we observe two dips (similar to the ones seen previously in photocurrent spectra of bulk TMDCs^40^) at ~1.9 eV and ~2.1 eV (Fig. 1c) on top of a largely featureless device-dependent background photocurrent. Upon annealing, this background, attributable to absorption by midgap states^41^ as well as to photogating artifacts^42^,^43^ (Supplementary Information, S3) recedes leaving a set of universal features seen in every device. We note that some devices do not require annealing and exhibit clean PC spectrum right after suspension. Photoconductivity spectrum of a high-quality device #2 is shown in Fig. 2a. We observe: (*i*) Two sharp peaks at ~1.9 eV and ~2.1 eV (labeled ‘A’ and ‘B’, respectively), (*ii*) near-zero PC signal below the A-peak, between A- and B-peaks and above the B-peak (from ~2.1 eV to ~2.5 eV), (*iii*) steep growth of PC starting at ~2.5 eV, and (*iv*) a broad and strong peak ‘C’ at ~2.9 eV. To the best of our knowledge, this is the first observation of the features *(ii)–(iv)* in PC spectroscopy. Next, we demonstrate that all of these features originate from optical absorption by bound excitons as well as by unbound electron-hole (*e–h*) pairs in MoS_2_.

Features A and B stem from optical absorption by the well-known^5^,^7^,^34^ A- and B- band edge excitons of MoS_2_ residing at *K*-points of the Brillouin zone (Fig. 1d, Inset). Recombination of these excitons results in photoluminescence peaks at similar spectral positions (Fig. 1d). The ~160 meV separation between the A- and B- peaks is a consequence of the splitting of the valence band of MoS_2_ at the *K* point due to spin-orbit interactions^5^,^6^,^7^. The positions of the A- and B-peaks are also in good agreement with the calculated optical spectrum that we obtain using first-principles GW-BSE calculations (Fig. 2c, light-red curve,)^27^,^44^,^45^,^46^. See Supplementary Information, S6 for details.

The feature at ~2.9 eV (‘C’) has been previously noted in absorption spectrum of MoS_2_^5^,^34^,^44^, but to the best of our knowledge not thoroughly analyzed. We interpret this peak as coming from an excitonic state associated with the van Hove singularity of 1L-MoS_2_. This van Hove singularity is peculiar, as neither the conduction nor the valence bands have singularities in the density of states in the corresponding region of the Brillouin zone between *K* and *Γ* points (orange curves in Fig. 2b and Fig. 2d). At the same time, the bands are locally parallel in that region, causing a local minimum in the Mexican-hat-like *optical band structure* (difference between conduction and valence bands shown in Fig. 2b as red and green curves). This minimum is prominent in a 2D colorplot of the optical band structure as a continuous gear-shaped region circling the *Γ* point (Fig. 2e, dark red region). The large joint density of states associated with this minimum yields a strong peak in 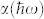. Indeed, our GW calculations (*i.e.*, without inclusion of excitonic effects) of the optical spectrum prominently feature a sharp peak at ~3.45 eV, the value that corresponds to the optical band gap at the van Hove singularity point (Fig. 2c, black curve). Excitonic effects downshift the peak to ~2.9 eV (Fig. 2c, light-red curve), very close to the experimentally measured position of the C-peak. Interestingly, the C-exciton valley of the optical bandstructure is near-rotationally symmetric rendering this exciton effectively one-dimensional^47^. Moreover, the location of the C-exciton at the bottom of the Mexican hat dispersion suggest that this exciton is localized in both real and momentum space, a conclusion also supported by first-principles calculations^26^,^44^.

Within the resolution of our measurements (signal-to-noise ratio is ~20 for A/B-peaks), we observe zero photocurrent below the A-peak, between the A- and B-peaks and between the B- and C-peaks. This observation is in contrast with non-zero optical absorption^5^ and photocurrent in the same region in supported devices measured by us (data in the Supplementary Information, S4) as well as by others^5^,^34^. It has been previously suggested^48^ and observed^34^,^49^ that disorder-related midgap states can significantly perturb the optical response of MoS_2_ leading to below-band gap absorption. Moreover, reduction in the background absorption upon annealing, which is likely associated with reduced disorder, has been recently observed in chemically exfoliated MoS_2_ samples^34^. We therefore interpret the lack of PC background in our devices as a signature of the low density of the disorder-related midgap states. Moreover, we do not observe any features due to trions^12^,^13^ and trapped excitons^49^, which suggests that our devices are undoped and contain low defect density. We also note that despite the high quality of our devices, no signatures of anticipated^26^,^50^ excited states of A- or B-excitons are observed. This is consistent with the very low oscillator strength of these states expected from a simple 2D hydrogen model (see Supplementary Information, S7).

Above the near-zero photocurrent region, we observe a featureless and abrupt increase of the PC above *E_g_^exp^* ~ 2.5 eV. This increase is clearly visible in the plot of 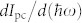 (Fig. 2a, Inset). The PC onset occurs very close in energy to the calculated *fundamental* (i.e. single-particle) band gap of 1L-MoS_2_, *E_g_^calc^* ~ 2.55 eV (Fig. 2b–c) and is therefore related to direct band-to-band absorption by unbound *e–h* pairs. However, experimentally we cannot distinguish the onset of the band-to-band absorption from the tail of the C-peak. We therefore interpret that the measured value of *E_g_* is a lower bound for the fundamental band gap value. We can therefore *experimentally* estimate the exciton binding energy in MoS_2_ as *E_bind_ = E_g_ − E_A_* ≥ 570 meV. We emphasize that in our suspended devices the measured values for *E_g_* and *E_bind_* are free from the influence of the substrate-related dielectric screening and hence can be directly compared to calculations (Fig. 2a–c).

We now turn to bi- and multi-layer MoS_2_, as well as other 1L-TMDCs, such as MoSe_2_ and WSe_2_. Similar A-, B-, and C- features are seen in photocurrent spectra for all of these materials (Fig. 3a). For materials other than 1L-MoS_2_, however, we do not observe the zero photocurrent between B- and C-peaks. This precludes direct experimental estimation of exciton binding energies in these materials. However, since our first-principles calculations of *E_g_*, A-, B- and C-peaks for 1L MoS_2_ are in good agreement with the experimental data, we can infer *E_g_* and *E_bind_* of other TMDC materials from corresponding A-, B- and C-peak positions (details are in Supplementary Information, S6). We note the following trends:

- The A- and B- peaks in MoS_2_ do not depend significantly on its thickness (Fig. 3b, red points)^5^. This is a consequence of simultaneous and nearly equal reduction of *E_g_* (Fig. 3b, black points) and *E_bind_* with the number of layers of MoS_2_^51^.
- The splitting between A- and B- peaks is largest in WSe_2_ (~510 meV), followed by MoSe_2_ and MoS_2_ (Fig. 3d). This is a signature of the stronger spin-orbit interaction in WSe_2_, related to the higher atomic number of tungsten.
- The calculations suggest that variation of the type of chalcogen (S, Se) atom has a strong effect on *E_g_* (Fig. 3c). This is a consequence of the dependence of the lattice constant on the type of chalcogen atoms. On the other hand, *E_bind_* remains roughly constant for all measured materials (Fig. 3d).

Our next aim is to understand very large PC magnitude. To contribute to photocurrent, a neutral exciton must first dissociate into an unbound electron-hole pair. This process is characterized by the probability *D* entering into Eq. (1). To investigate the mechanism of dissociation in 1L-MoS_2_, we examine *I_PC_* *vs.* *V_ds_*. We find that the A- and B- peaks in the photocurrent practically disappear at low *V_ds_*, while the C-peak remains prominent (Fig. 4a). This behavior is consistent with dissociation of excitons by strong electric fields arising near the interface between MoS_2_ and metallic contacts. Indeed, a large electric field is required to overcome the binding energy *E_bind_* ≥ 0.6 eV for A-excitons. Such a field can arise at the interface between MoS_2_ and a metallic contact due to the application of a large bias voltage (like in the case of pristine organic semiconductors^52^) and possibly due to the mismatch of the work functions of MoS_2_ and metal (similar to nanotube devices^53^ and excitonic solar cells^54^). Our conclusion that PC is produced only at the contacts is also supported by scanning photocurrent microscopy measurements directly mapping photocurrent production^23^. In contrast, C-excitons exist above the band gap and therefore can produce unbound *e–h* pair even without application of an external electric field. Thus we demonstrate for the first time electric field assisted dissociation of A- and B-excitons and spontaneous decay of C-excitons into a free electron-hole pairs.

Finally, we analyze the reason for the very large photogain (*η* > 1,000) and photoresponsivity (~50 A/W) in our devices (Fig. 4b). Photoresponsivity ranging from ~1 mA/W^23^,^24^ to ~900 A/W^21^ has been previously reported for monolayer MoS_2_ and from ~5 mA/W^23^ to ~0.6 A/W^22^ – for multilayer MoS_2_. Previously suggested mechanisms, such as the direct dissociation at the contacts (yielding *η* < 1)^23^ or photothermoelectric effect (yielding *η* ≪ 0.1)^38^ cannot explain very high observed photogain. Generally, large gain can be related to multiplication of photocarriers due to the avalanche effect^35^. It can also originate from long photocarrier lifetime *τ* due to the trapping of photoexcited carriers either in the defect states (persistent photoconductivity^35^) or in the band-bending region between a metal contact and a semiconductor^55^. However, as mentioned above, clean suspended MoS_2_ devices only start to conduct (*G* ~ 10^−7^ S) at large (*V_ds_ > E_g_*/*e*) source-drain bias (Fig. 1b). Operation in this regime may be complicated by additional effects, such as Zener or thermal breakdown^55^. On the other hand, we observe that glass-supported MoS_2_ devices (chosen to eliminate parasitic photogating) have dark conductance *G* ~ 10^−5^ S, likely due to the higher doping level of supported MoS_2_. In agreement with Eq. (1), the photoresponse of these devices is correspondingly higher and can be observed even at small *V_ds_* (Fig. 4b). Moreover, the relatively low resistance and correspondingly low RC time-constant of glass-supported devices allows us to measure the time dependence of the photocurrent.

The observation of *η* ~ 25 at *V_ds_* ~ 0.5 V for a glass-supported device #4 (Fig. 4b) rules out the avalanche effect as the mechanism responsible for the observed high photogain. In this regime, the energy *eV_ds_* is well below the fundamental band gap and is not sufficient to start an avalanche. Persistent photoconductivity has been previously reported in MoS_2_^21^, but we can exclude it as a possible candidate for the PC generation in clean MoS_2_ because we routinely observe characteristic photoresponse time < 1 ms at low temperatures (Fig. 4c). This is approximately five orders of magnitude faster than the response time reported for persistent photoconductivity^21^, but still slower compared to the carrier transit time (time it takes a carrier to travel across the device). The large photogain of our devices is most consistent with photocarrier trapping mechanism also seen in metal-semiconductor-metal and tunnel-emitter phototransistors^55^. Upon illumination, photoexcited holes are trapped in the potential well formed due to band bending^56^ at the interface between MoS_2_ and Au metallic contacts. At the same time, the electrons are injected into the MoS_2_ channel (Fig. 4d). According to the Eq. 1 this leads to very large changes in the conductivity. First, spatial separation of photocarriers precludes their recombination and greatly increases their lifetime *τ*. Second, high concentration of holes near the metal-semiconductor junction decreases the thickness of the Schottky barrier and reduces the contact resistance^55^.

In conclusion, we note several potential applications of the obtained results. First, the large photogain, fast photoresponse, and bias-voltage dependence of the photocurrent spectra of pristine monolayer TMDCs suggest applications of these materials as sensitive and voltage-tunable photodetectors^57^. Second, the high absorption and dissociation probability of C-excitons may be employed in creating efficient TMDC-based solar cells^25^,^58^. Finally, our study confirms that the properties of TMDCs are strongly affected by their environment. This may prove important in designing TMDC-based electronic and optoelectronic devices.

*While the manuscript was under review, several groups reported measurements of the binding energy for excitons in 1L TMDCs. Some studies obtained the binding energy ~ 600–700 meV^59^,^60^,^61^, which is very close to our estimates. Others observed lower binding energy ~ 350 meV^62^,^63^ for substrate-supported devices, but predict values close to ~500 meV for suspended devices^62^.*

## Author Contributions

A.R.K. and A.K.M.N. prepared the samples, performed the experiment and analyzed the data; K.I.B. supervised the project; B.W. and S.T.P. conducted the first-principles calculations; J.L. and S.T.P. conducted the STEM imaging; K.A.V. performed analytical calculations; D.P. prepared the glass-supported TMDC samples; A.R.K., A.K.M.N., H.K., B.L.I. and N.H.T. designed and built the spectroscopic measurement unit; D.C., A.B., N.J.G., J.Y. and D.G.M. grew the TMDC bulk crystals; A.R.K., A.K.M.N. and K.I.B. co-wrote the manuscript with input from all authors. All authors discussed the results.

## Supplementary Material

Supplementary Information Supplementary Information

## Figures and Tables

**Figure 1:**
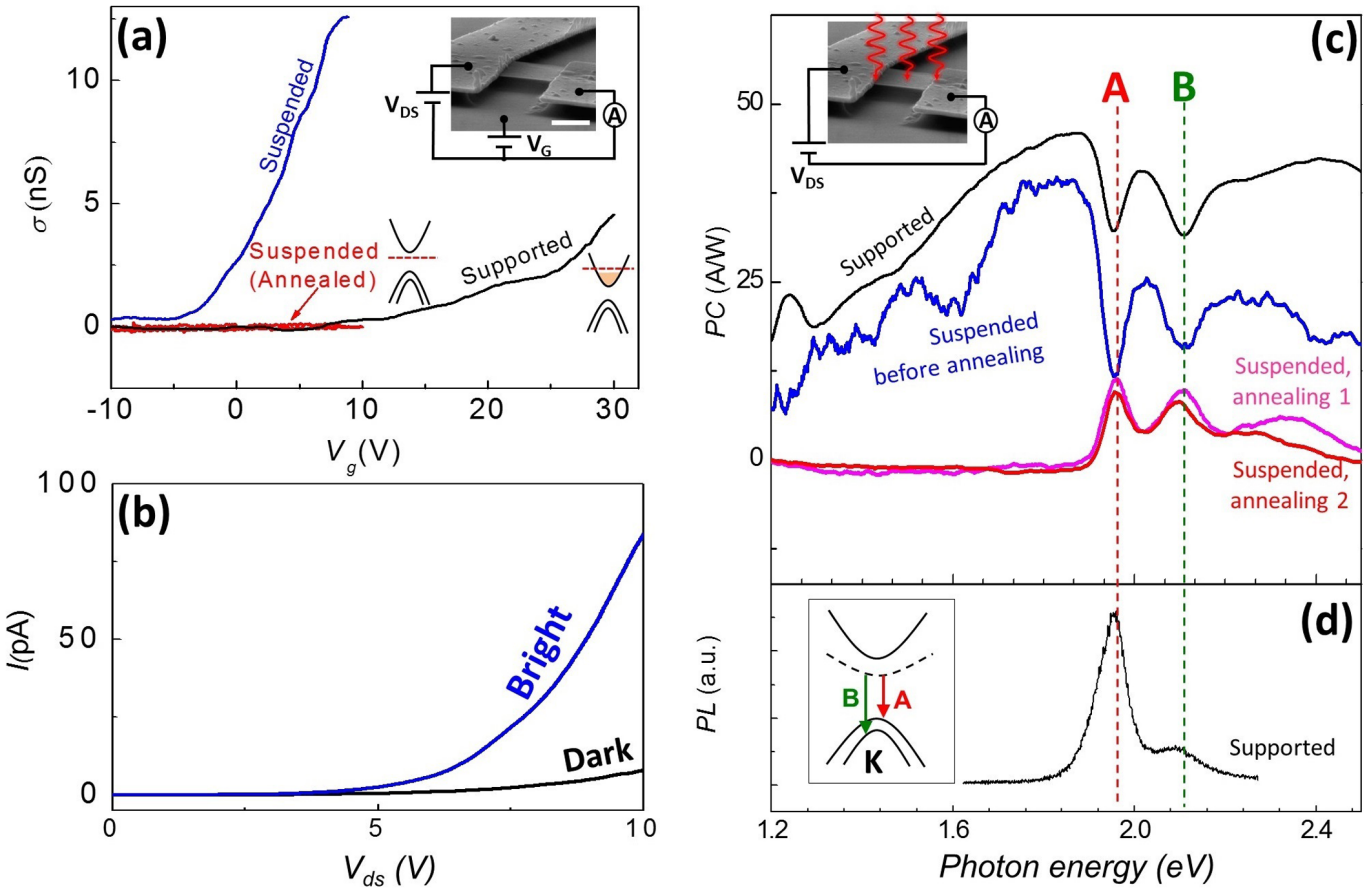
Effects of substrate and thermal annealing on conductance and photocurrent of suspended MoS_2_. (a) Gate-dependent conductance of supported, suspended, and suspended annealed 1L-MoS_2_ device #1 at *T* = 300 K. Inset: Image of the device. The scale bar is 1 μm. Schematically drawn band diagrams show the position of the Fermi level (red dashed line). (b) Dark and bright electrical response of an annealed suspended device #2 at *T* = 77 K. Illumination intensity is ~3 pW/μm^2^ and wavelength is *λ* = 430 nm. (c) Photocurrent (PC) spectrum of a supported and suspended MoS_2_ device #1 at different stages of thermal annealing at *T* = 77 K. (d) Photoluminescence spectra for a supported MoS_2_ device #1 at *T* = 300 K. Since PL spectra were recorded at room temperature, we manually blue-shift them by 150 meV to allow comparison with PC spectra obtained at *T* = 77 K (see Supplementary Information, S5 for details). Inset: Bandstructure schematics of MoS_2_ near *K*-point illustrating the origin of band-edge excitons. The dashed line represents excitonic states.

**Figure 2:**
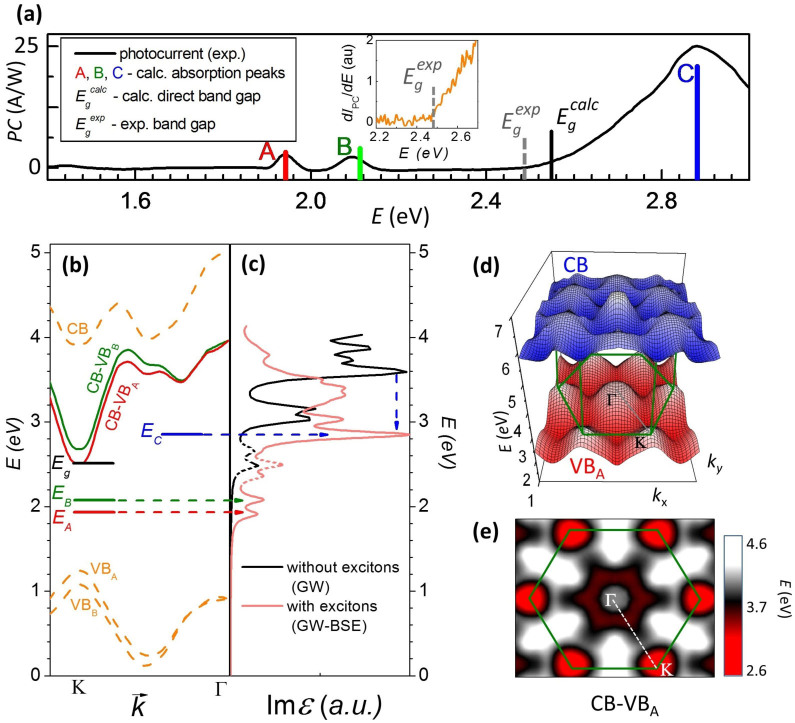
Probing excitons in pristine monolayer MoS_2_ through photocurrent spectroscopy. (a) PC spectrum of an intrinsic suspended unannealed 1L-MoS_2_ device #2 acquired at *V_ds_* = 6 V. Background photocurrent due to the surface photovoltage was subtracted (Supplementary Information, S3). Calculated positions of excitonic A-, B- and C-peaks and band gap *E_g_* are shown as colored vertical bars. The bar height represents peaks amplitudes. The inset: derivative of the photocurrent plotted vs. the photon energy. (b) Electronic and optical band structures of 1L-MoS_2_ along the *K-Γ* direction. The solid horizontal lines are the estimated positions of the excitonic bound states. (c) Optical spectrum of MoS_2_ calculated with and without excitonic effects. The dashed peaks between 2.2 eV and 2.7 eV are computational artifacts, which are discussed in the Supplementary Information, S6. Vertical blue arrow indicates the position of the van Hove singularity downshifted by excitonic effects. (d) Three-dimensional plot of the band structure of MoS_2_. (e) The colorplot of the optical band structure of MoS_2_. Dark red gear shaped region around *Γ* is the local minimum corresponding to the excitonic C-peak.

**Figure 3:**
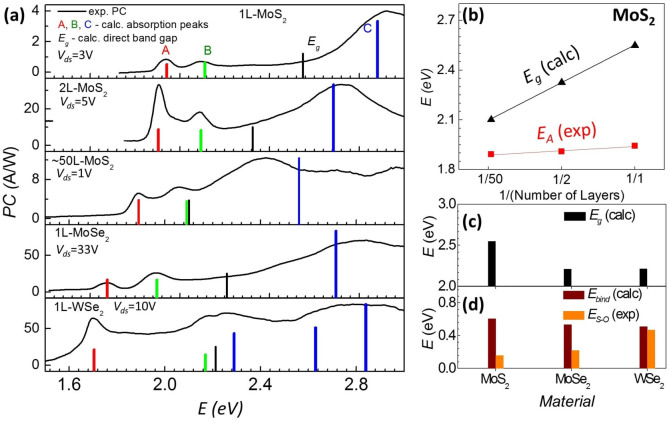
Photocurrent in various TMDC materials. (a) Experimental PC spectra of different TMDC devices. All the devices are suspended and annealed except for the multilayer MoS_2_ device, which is supported on a glass substrate (Supplementary Information, S1). 1L MoS_2_ sample is an annealed device #3. For each device, the bias voltage was chosen to maximize the signal to noise ratio for the photocurrent. Solid bars are calculated excitonic peaks and band gap values. Large spin-orbit coupling of WSe_2_ results in splitting of the valence and the conduction bands even near *Γ*-point, which leads to splitting of the C-peak. (b) Dependence of excitonic peak positions and band gap values on number of layers of MoS_2_. (c,d) Comparison of *E_g_*, *E_bind_* and spin-orbit coupling strengths for different 1L-TMDCs.

**Figure 4:**
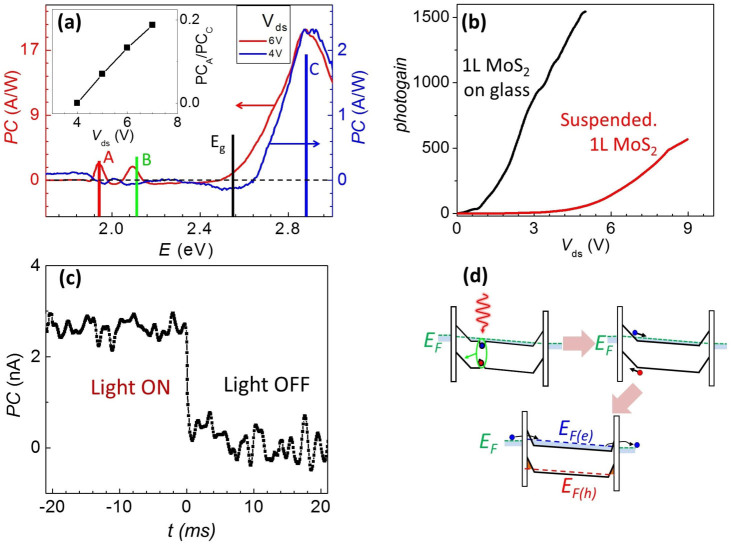
Photoconversion mechanisms in monolayer MoS_2_. (a) PC spectra measured in a suspended 1L-MoS_2_ at two different *V_ds_*. Both curves are normalized to the height of the C-peak. Inset: relative PC amplitudes of A- and C- peaks *vs.* *V_ds_*. Note that apparent negative photocurrent around ~2 eV and ~2.5 eV is an artifact caused by our procedure for background subtraction (Supplementary Information, S3). (b) Photogain for a glass-supported and suspended devices *vs.* *V_ds_*. The device is illuminated at λ = 640 nm with intensity ~ 30 pW/μm^2^. (c) Time response of PC to the varying light intensity in a glass-supported MoS_2_ (device #4) This measurements sets the upper limit for the response time < 1 ms. Accuracy of time-resolved measurements was limited by the high resistance of MoS_2_ and therefore high RC-time constant of the measurement circuit. (d) Schematics (not to scale) of the photogain mechanism. *E_F_*, *E_F(e)_* and *E_F(h)_* represents the Fermi level, and quasi-Fermi levels for electrons and holes respectively.
