# Supplementary material for: Probing excitonic states in suspended two-dimensional semiconductors by photocurrent spectroscopy
Source: Sci Rep. 2014 Oct 16;4:6608. doi: 10.1038/srep06608 (PMC4198869; doi:10.1038/srep06608)
Supplement: Supplementary Information [file srep06608-s1.pdf]

Supplementary Information

Probing excitonic states in suspended two-dimensional semiconductors by photocurrent spectroscopy

A. R. Klots[1]*, A. K. M. Newaz[1]*, Bin Wang[1], D. Prasai[2], H. Krzyzanowska[1], Junhao Lin[1,4], D. Caudel[1], N. J. Ghimire[3,4], J. Yan[4,5], B. L. Ivanov[1], K. A. Velizhanin[6], A. Burger[7], D. G. Mandrus[3,4,5], N. H. Tolk[1], S. T. Pantelides[1,4], and K. I. Bolotin[1]

[1]Department of Physics and Astronomy, Vanderbilt University, Nashville, TN-37235, USA
[2]Interdisciplinary Graduate Program in Materials Science, Vanderbilt University, Nashville, TN-37234, USA
[3]Department of Physics and Astronomy, University of Tennessee, Knoxville, TN-37996, USA
[4]Materials Science and Technology Division, Oak Ridge National Laboratory, Oak Ridge, TN-37831, USA
[5]Department of Materials Science and Engineering, University of Tennessee, Knoxville, TN-37996, USA
[6]Theoretical Division, Los Alamos National Laboratory, Los Alamos, NM-87545, USA
[7] Department of Physics, Fisk University, Nashville, TN-37208, USA

(*These authors contributed equally to the work)

S1. Fabrication and measurement techniques

S2. Effects of laser assisted thermal annealing

S3. Intrinsic and extrinsic features in PC spectra

S4. Comparison of absorption and photocurrent spectra

S5. Temperature dependence of the excitonic peak positions

S6. Computational methods

S7. 2D Hydrogen model for band edge excitons

S1. Fabrication and measurement techniques

Sample Preparation

Monolayer and multilayer TMDC samples were mechanically exfoliated from bulk TMDC crystals onto heavily *p*-doped silicon substrates covered by a 280 nm of thermal oxide. The sample thickness was confirmed by Raman spectroscopy measurements[1]. The metal electrodes were patterned by using e-beam lithography followed by thermal evaporation of Cr (2nm)/Au (90nm). The samples on glass were prepared by first fabricating Cr/Au electrode on top of a clean glass substrate and then by mechanically transferring TMDC flakes onto the electrodes[2]. To create suspended devices, the sacrificial SiO_2 was removed by buffered oxide etchant. To avoid collapse of suspended devices, finished specimens were dried in a critical point dryer[3].

Optical measurements

The samples were measured in an optical cryostat at temperatures between 77K and 300K. For photocurrent spectroscopy, the optical beam from a thermal source (halogen lamp) was guided through a monochromator (Cornerstone, Newport Corp.) and a mechanical chopper onto the sample where it was focused down to ~2 mm^2 spot. To calibrate the light intensity at a sample, the intensity of the split beam was recorded by a Si detector (Thorlabs DET36A). Both photocurrent and light intensity were measured simultaneously using a lock-in technique.

Annealing of suspended devices

Suspended monolayer devices are loaded into an optical cryostat with a base temperature ~77K. The devices are heated by an infrared (wavelength 10.6 μm) laser beam, while the cryostat is kept at its base temperature. To avoid structural and chemical modification of the sample, the laser beam is defocused into a ~0.5 mm^2 spot, so that the power flux on the sample is relatively low ($<20 \mu W/\mu m^2$). We estimate that this procedure allows heating the die containing suspended devices by ≤500K.

During the process of annealing, the device is heated gradually by slowly increasing the laser power, and then measured using PC spectroscopy. The process is repeated until the background photocurrent below the A peak is no longer observed. We note that rarely (<5% of all measured devices), we observe signatures of low disorder – zero background PC below the A peak and between B and C peaks in **unannealed** devices. Spectra and electro-optical characteristics of one such device are presented in Fig. 2a and Fig. 4a-b. Similarity between PC spectra of clean unannealed (Fig. 2a) and clean annealed (Fig. 3a) devices proves that laser annealing does not chemically modify or damage the suspended monolayer materials.

For multilayer MoS_2 devices, suspension and annealing do not significantly reduce broad PC background. Because of this, the data reported in the main text (Fig. 3a) are collected using a device supported on a glass substrate. Due to their low surface-to-volume ratio, thick multilayer (>50L) flakes are not strongly affected by the environment. Therefore, we believe that the optoelectrical properties of such devices are close to intrinsic.

Supported devices on glass

To prepare samples on glass substrates, we first pattern Cr/Au electrodes on top of a microscopy glass slide (Corning Boro-Aluminosilicate, Delta-Technologies Limited). We start the preparation of the glass slides by cleaning with piranha solution (67% H_2SO_4 and 33% H_2O_2). After deposition of the electrodes, the substrate is again cleaned in piranha solution followed by reactive ion etching. Flakes of TMDC materials are then mechanically transferred onto electrodes[2].

Lock-in measurements

In our photocurrent measurements, monochromated light from a thermal source chopped at 930 Hz is used for illumination. The constant source-drain bias V_{ds} up to 20 V is applied across the device. The absolute magnitude of photocurrent is recorded by measuring the source drain current with a lock-in amplifier. The lock-in phase is optimized at the A-exciton peak position. To remove the features due to artifacts, photocurrent at V_{ds}=0 is subtracted from the measured data (see below, section S3). Finally, the photocurrent is normalized to the illumination intensity (see below).

Normalization of the photocurrent spectra

To account for the variation of the spectral power density of the tungsten-halogen light source, we normalize the recorded PC spectra to the illumination power. For monolayer and bulk TMDC devices, the PC response is proportional to the illumination power and the procedure is justified (Fig. S1). For bilayer MoS_2 devices, however, the dependence between PC and illumination power is non-linear for the illumination intensity that we use. Because of this, we did not normalize the PC spectrum for a bilayer MoS_2 device that is shown in Fig. 3a. Non-uniform spectral density of the illumination source can significantly distort relative heights of A-, B- and C- peaks, but not their positions. Indeed, measured positions of the A and B peaks for bilayer MoS_2 are in close agreement with the positions of the peaks in optical absorption reported by Mak *et al.*[4].

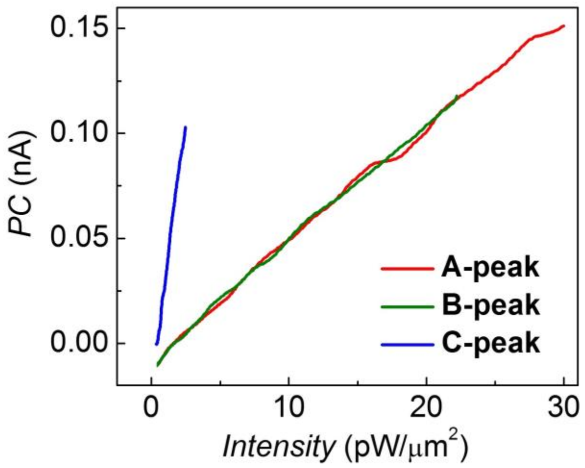

Figure S1. Power dependence of the photocurrent in 1L MoS_2 device. Measurements were performed for the three observed peaks: A-peak at ~1.9 eV, B-peak at ~2.1 eV and C- peak at ~2.9 eV. We note that the photosensitivity at the C-peak wavelength is an order of magnitude larger than that for A- and B- peaks.

Device Statistics

Table 1 presents the overall statistics of measured devices. In this table, the number outside the parenthesis is the number of successful devices, defined as the devices for which the PC spectra exhibits clearly resolved A-, B- and C-peaks with zero background below the A-peak. The number inside the parenthesis is the total number of fabricated devices.

Table 1: Device Statistics

	1L MoS_2	2L MoS_2	Multilayer MoS_2	1L $MoSe_2$	1L WSe_2
Suspended on Si/SiO_2 wafer	6(44)	5(37)	0(16)	2(16)	1(20)
Glass supported	6(6)	1(1)	5(5)	-	-

Typical dimensions of our devices are: 1 μm × 1μm for suspended MoS_2 devices and 1 μm × 2μm for glass-supported devices, 0.5μm × 1μm for $MoSe_2$ and WSe_2 devices.

S2. Effects of laser assisted thermal annealing

To elucidate the effect of the laser annealing on the structural properties of monolayer MoS_2, we have conducted scanning transmission electron microscopy (STEM) imaging, Raman, and PL spectroscopy of suspended 1L MoS_2 before and after annealing.

STEM imaging of laser annealed 1L MoS_2

We performed aberration-corrected atomically resolved STEM imaging of annealed and unannealed suspended MoS_2 samples. We transferred monolayer MoS_2 onto 3 μm holes in a gold-coated thick Si_3N_4 membranes. We then took these devices through the same annealing procedures as the devices studied in our manuscript. Our data confirm that concentration of defects in our samples after annealing is very low. Unfortunately, the high quality of our samples makes it difficult to quantitatively characterize changes in defect concentration upon annealing. Also, since the samples were prepared at Vanderbilt, then transported to and imaged at the Oak Ridge National Laboratory, ambient contamination was unavoidable. At the same time, STEM images shown in Fig.S2 unequivocally show that our annealing procedure does not introduce additional defects in MoS_2.

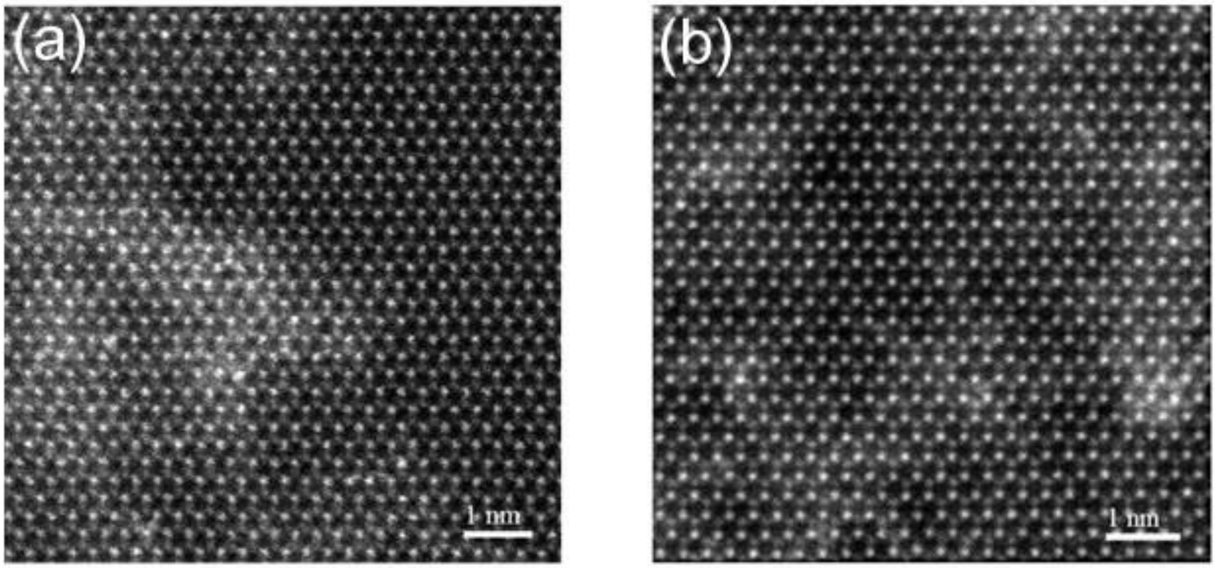

Figure S2: STEM images of unannealed (a) and annealed (b) monolayer MoS_2.

Raman spectroscopy of annealed samples

We conducted detailed Raman spectroscopy of suspended MoS_2 samples before and after annealing. Upon annealing, we observed ~1cm^{-1} blue-shift in Raman spectrum of MoS_2 as shown in Fig.S3. These shift cannot be attributed to the changes in mechanical strain[5]. According to a recent work by Chakraborty *et al.*,[6] such changes in the Raman peak positions are consistent with a ~3x10^{12} cm^{-2} decrease in the doping level of the device upon annealing.

PL spectroscopy of annealed samples

We compared room-temperature photoluminescence (PL) spectra of suspended MoS_2 samples before and after annealing. In some samples, we observed ~20% narrowing of the main *A*-peak upon

annealing as shown in Fig. S3b. The reduction in the width of PL peaks is typically associated with reduced disorder.

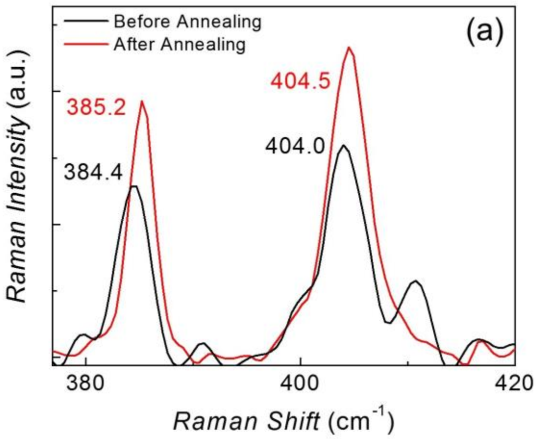

Fig.S3: a) Raman spectrum of suspended flake before (black) and after (red) annealing. Peak positions are marked by red and black numbers and are obtained by Lorentzian fitting. b) Normalized PL spectra of the same device before (black) and after (red) annealing.

Electrical and optoelectronic measurements

We measured electrical conductivity and photocurrent spectra of MoS_2 during annealing (Fig.S4). We typically observe gradual decrease of conductivity and gate efficiency during annealing (Fig.S4a). As devices are annealed step-by-step, we observe a strong decrease in quasi-random background features in the photocurrent spectra as shown in Fig.S4b, while leaving behind cleanly resolved universal features seen in every devices – A-, B-, and C-excitonic peaks.

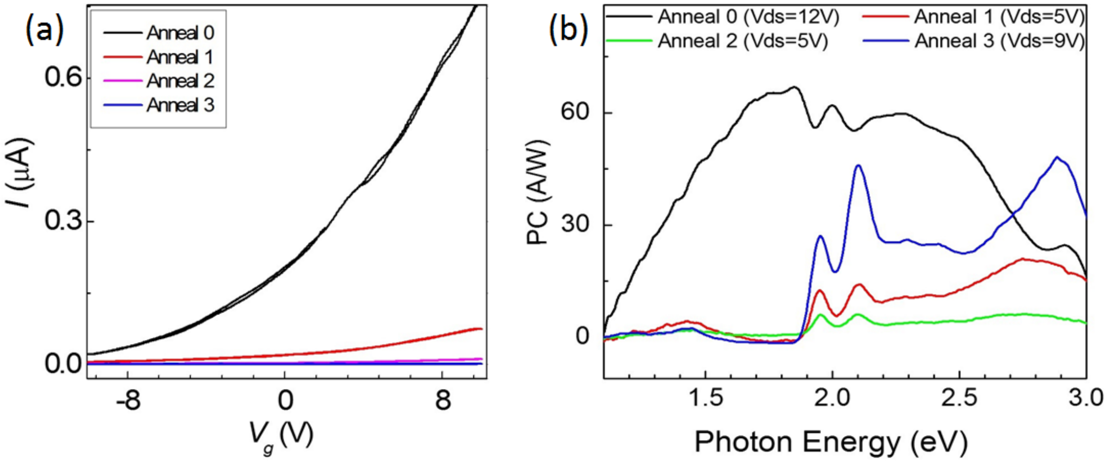

Figure S4: Electrical transport (a) and photocurrent spectra (b) in a suspended monolayer MoS_2 device at different stages of the annealing. Device before annealing is labeled 'Anneal 0'.

S3. Intrinsic and extrinsic features in PC spectra

Photocurrent spectra of some devices show features that vary from device to device and hence cannot be interpreted as intrinsic spectral features of TMDCs. In this section, we analyze various mechanisms that produce artifacts in PC measurements. First, in supported and suspended devices before annealing (Fig.1c, Fig. S5), we observe broad featureless photocurrent spectrum with two dips (at ~1.9 eV and ~2.1 eV). The amplitude of the background photocurrent grows monotonically with V_{ds}. We attribute

this background PC response to photo-field-effect (PFE)[7]. Second, in devices on Si/SiO_2, we observe non-zero photocurrent at zero V_{ds} when lock-in measurements are used (Fig.S5a-b). This photocurrent signal at V_{ds}=0 is also largely featureless. We attribute this signal to displacement photocurrent[8]. Finally, sometimes we observe photocurrent between A and B peaks and above the B peak in devices where PFE and displacement photocurrent are not present (Fig.S5c). This photoresponse is likely due to **absorption by midgap states**[9-11] of TMDCs. Next, we will discuss the details of each of these mechanisms and outline the procedures for removing them.

Photo-field-effect (PFE)

Current across a device under illumination is given by:

$$I = V_{ds}G(V_{ds}, n) \quad \text{(S1)}$$

, where G is the device conductance, and n is the number of carriers (see derivation of the Eq. (1) of the main text). In interpreting the PC spectroscopy data, we typically assume that the photocurrent originates from light-induced creation of additional photoexcited carriers inside a device. However, there is another less obvious way through which light can also produce changes in I. This mechanism, typically called photo-field-effect (PFE) or photogating effect[7], is effective when a light-absorbing semiconductor, such as Si substrate, is present close to a device. When the device is illuminated, photocarriers inside Si produce surface photovoltage at the Si/SiO_2 interface[12]. The resulting electric field effectively gates devices causing changes in n and hence photocurrent. The following evidence indicates the contribution of photo-field-effect in our experiments:

1) As expected for PFE, the photocurrent response in TMDC devices on Si/SiO_2 features a broad background starting at ~1eV, which is the absorption edge of Silicon (Fig.S5a).
2) The amplitude of this background PC grows monotonically with V_{ds}, as expected for PFE (Eq. (S1)).
3) The background PC below the A-exciton vanishes completely for devices without light-absorbing substrate, such as MoS_2 devices on glass (Fig.S5c).

We note that photo-field-effect also disappears for suspended devices upon annealing (Fig.S5b). We do not currently understand the mechanisms of this effect.

Combined contribution of the intrinsic photoresponse of MoS_2 and PFE can explain why A- and B-excitonic states in supported and most suspended but unannealed devices appear as dips rather than peaks in PC measurements (Fig.1c). According to the study of the photo-field-effect by Freitag *et al.*[7], photogating arises due to band-bending on the interface between Si and SiO_2. In the case of our *p*-doped wafers (instead of *n*-doped wafer used by Freitag *et al.*), we expect downward band-bending, which should lead to accumulation of electrons at the Si/SiO_2 interface. These electrons act as an additional negative source of gate voltage decreasing the conductivity of MoS_2. On the contrary, direct absorption of light increases conductivity of MoS_2. Our lock-in measurements used in recording PC are only sensitive *to the absolute value of the total photocurrent*. If PFE signal is stronger than the intrinsic MoS_2 photoresponse, resulting measured current is $|I_{result}|=|I_{intrinsic}-I_{PFE}|= |I_{PFE}|- |I_{intrinsic}|$. In this situation, we expect to see excitonic photocurrent to appear as dips, rather than peaks, in agreement with the experimental measurements as shown in Fig.1c. We note that similar dips in photocurrent at the spectral positions of the A- and B- excitons have been previously observed in multilayer TMDCs by Kam *et al.*[13] However, the mechanism we propose here is different from that of Kam *et al.*

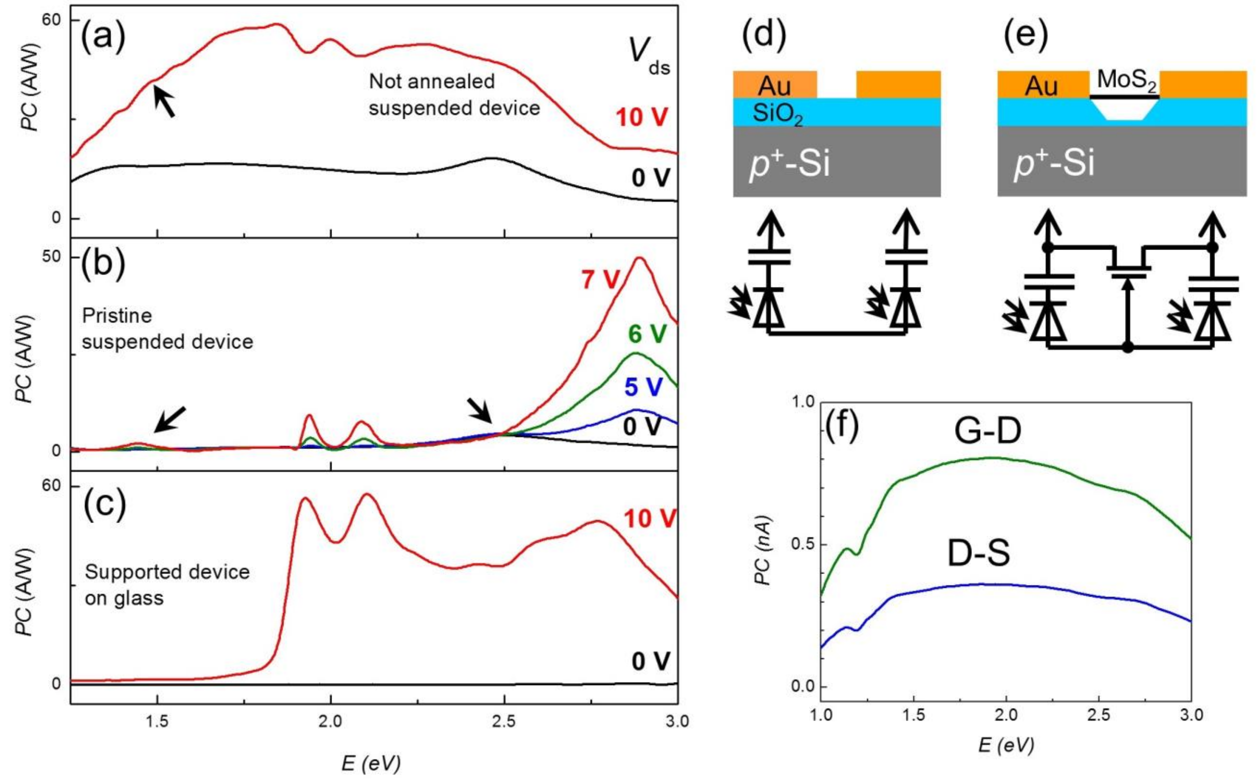

Figure S5: *Artifacts in PC of MoS_2-devices. (a)-(c) Photocurrent spectra for MoS_2 devices in different conditions. (a) Strong featureless background PC in suspended unanneled device due to photogating. (b) Pristine suspended unannealed device shows response at zero source-drain bias (black curve). (c) Device on glass shows no substrate-related artifacts, but has response in the region 2.0 eV-2.5 eV, likely due to the absorption by midgap states. (d) Structure and schematic representation of an empty device. Photodiodes symbolize photogeneration of surface charge on Si/SiO_2-interface and capacitors indicate the capacitance between Au electrodes and Si-substrate. (e) Structure of MoS_2-photodetector on Si-Substrate and its schematic representation. MoS_2 is represented as a field-effect-transistor, gated by the surface photovoltage on Si/SiO_2 interface. (f) Photoresponse of an empty device. Green curve: PC measured between gate and drain (G-D). Blue curve: PC measured between source and drain (D-S).*

Photoconductivity due to electron-exciton scattering

Below, we roughly estimate the impact of electron-exciton scattering on device conductivity. Assuming exciton lifetime of τ=10ps[14,15], absorption coefficient of MoS_2 α=0.1[4], photon energy E_{ph}~2eV and illumination intensity P=30pW/µm^2 (typical conditions in our experiments), we estimate the density of photogenerated excitons $n=\alpha P \tau / E_{ph} \sim 10^4$ cm^{-2}. This density implies ~100 µm separation between excitons, which is ~100 times bigger than our device dimensions. This means that at any point of time there is, on average, much less than one exciton in our device. *We therefore conclude that the presence of excitons does not affect carrier mobility.* At the same time, the lifetimes of photoexcited charge carriers are many orders of magnitude longer than 10ps (Fig.4c gives an upper bound for this lifetime, <1ms), and the density of photoexcited carriers is correspondingly higher.

Displacement photocurrent

For some devices on Si/SiO_2 wafers, we observe photocurrent even at zero source-drain bias (Fig.S5a-b). Moreover, similar PC response is observed for a test device that does not contain a conductive TMDC layer between the electrodes (Fig.S5f). Clearly, this type of photoresponse cannot be attributed to photo-field-effect and is not an intrinsic response of TMDCs. We propose that this photoresponse originates from the displacement photocurrent between Si and metal electrodes[8]. Indeed, our lock-in measurements of the photocurrent employ a light source with time-varying intensity. This produces a time dependent surface photovoltage of Si, and therefore time dependent potential difference across capacitor formed between Si back-gate and electrodes. Slight asymmetry between source and drain electrodes leads to the appearance of the displacement current between the electrodes. The following experimental evidence is consistent with the contribution due to displacement photocurrent:

1) Similarly to the case of photo-field-effect, displacement photocurrent vanishes for MoS_2 devices supported on glass (Fig.S5c, black curve). This is expected, as these devices do not contain silicon substrate.
2) Small illumination power and, consequently, negligible density of excitons in our devices rules out electron-exciton scattering.
3) As expected for displacement current, the phase of the PC at zero bias is shifted by ~90° with respect to the illumination signal.
4) In addition to negative photoconductivity for devices supported on Si/SiO_2, we typically observe broad background photoconductivity extending down to ~1eV, significantly below the optical bandgap of MoS_2 (~1.9eV) (Fig.S5). This background has spectral features similar to the absorption spectrum of Si and the $I_{PC}(V_{ds})$is consistent with the effect of photogating of Si/SiO_2 interface.

Whereas photogating can be remedied by annealing a suspended device, displacement photocurrent is independent of the TMDC quality and cannot be completely eliminated by annealing. However, it can be removed by subtracting zero-V_{ds} PC spectrum from the spectrum obtained under non-zero V_{ds} bias.

Disorder-related photocurrent

As discussed above, both photo-field-effect and displacement photocurrent are fully suppressed in MoS_2 devices on glass. However, even in these devices, we observe the background PC response above the A peak that varies from device to device (Fig.S5c). At the same time, this background almost completely disappears in some suspended and annealed devices (Fig.2a). This suggests that this background signal is *not intrinsic* to MoS_2. We propose that it is related to the absorption of light by the midgap states in TMDCs. Indeed, both experimental[9-11] and theoretical[16] work showed that disorder may modify optical spectra of TMDC and cause below-bandgap absorption.

To suppress disorder-related PC in a suspended device, we use thermal annealing. As discussed in the main text, background PC below the A peak and between B and C peaks disappears (Fig.2a) upon annealing. This is consistent with reduction in disorder in thin TMDC films upon thermal annealing reported by Eda et al.[10]

S4. Comparison of absorption and photocurrent spectra

Devices on glass, which are described above, also allow us to compare our photocurrent spectra with absorption spectra of MoS_2 obtained by other groups[4]. The photocurrent spectrum of a glass-supported 1L MoS_2 device, as shown in Fig.S6, is very similar to the optical absorption spectrum of 1L MoS_2 devices measured under similar conditions by Mak *et.al*.[4] This suggests that the photocurrent spectrum is roughly proportional to optical absorption spectrum and probes similar transitions. This is consistent with our expectation that the photogain (See Eq.1 of the main text) does not strongly depend on wavelength.

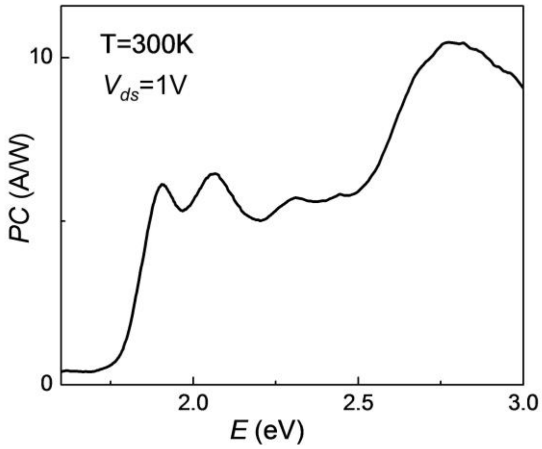

Figure S6: PC spectrum of a glass-supported 1L MoS_2 device at room temperature.

S5. Temperature dependence of the excitonic peak positions

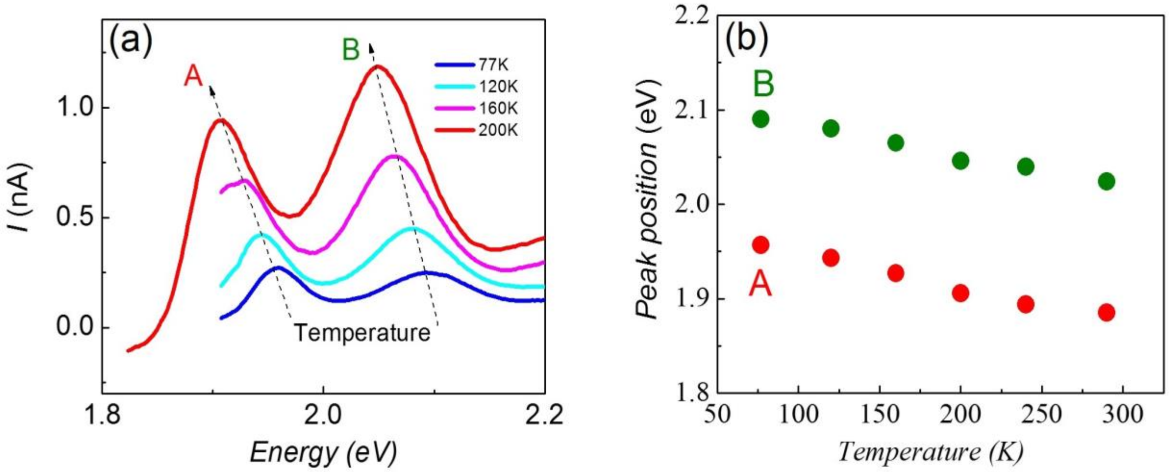

Figure S7: Temperature dependence of A- and B- peaks. (a) Spectra of a suspended 1L MoS_2 device at different temperature. (b) A- and B- peak positions of the same device as a function of temperature.

In our experiments, the positions of the A- and B- excitonic peaks in a suspended device blue-shift by ~150meV as we cool down the device from 300K to 77K (Fig.S7). Similar increase of MoS_2 bandgap with decreasing temperature has been previously reported[14]. In order to compare our PC spectrum recorded at 77K to the PL spectrum recorded at room temperature, we blue-shift the PL-spectrum by 150meV as in Fig.1e.

S6. Computational methods

Density functional calculations are performed using the VASP package[17]. The PBE-GGA exchange-correlation potential[18] is used and electron-core interactions are treated in the projector augmented wave (PAW) method[19,20]. The plane-wave kinetic-energy cutoff is set to 400 eV. The spin-orbit

coupling is taken into account. The layered MoS_2 is modeled using a supercell approach with adjacent layers separated by a vacuum region. The k-point mesh is set to 12 × 12 × 1 or higher (see the discussion below). The structures are optimized with all the atoms relaxed until the self-consistent forces reached 0.02 eVÅ^{-1}. The van der Waals interactions are taken into account using the semi-empirical correction scheme, the DFT+D approach[21].

The GW_0 calculations are performed using VASP[22]. The quasiparticle energies are obtained by iterating only Green function (G), but keeping screened Coulomb interactions (W) fixed to the initial DFT W_0. The energy cutoff for the response function is set to 100 eV to speed up the calculations, and about 100 empty bands are included in the calculations. The obtained quasiparticle energies are then employed to calculate the band structure using Wannier interpolation as implemented in WANNIER90 program[23]. Binding energies of the direct excitons are calculated by solving the Bethe-Salpeter equation (BSE) for the two-particles Green's function[24,25]. The calculations of BSE spectrum are carried out based on the Tamm-Dancoff approximation[26]. Eight highest valence bands and the eight lowest conduction bands are included in the calculation of the spectrum.

Multiple factors impact the precision of GW-BSE calculations. It has been shown[27] that for modeling mono- and few-layer TMDC materials, most relevant parameters are (i) k-point sampling of the Brillouin zone (BZ), (ii) vacuum layer thickness – spatial separation between two next neighboring TMDC layers (effectively, the height of a supercell), and (iii) number of GW-iterations. Unfortunately, limitations of the computational resources make it impractical to achieve convergence of the spectrum with respect to all computational parameters.

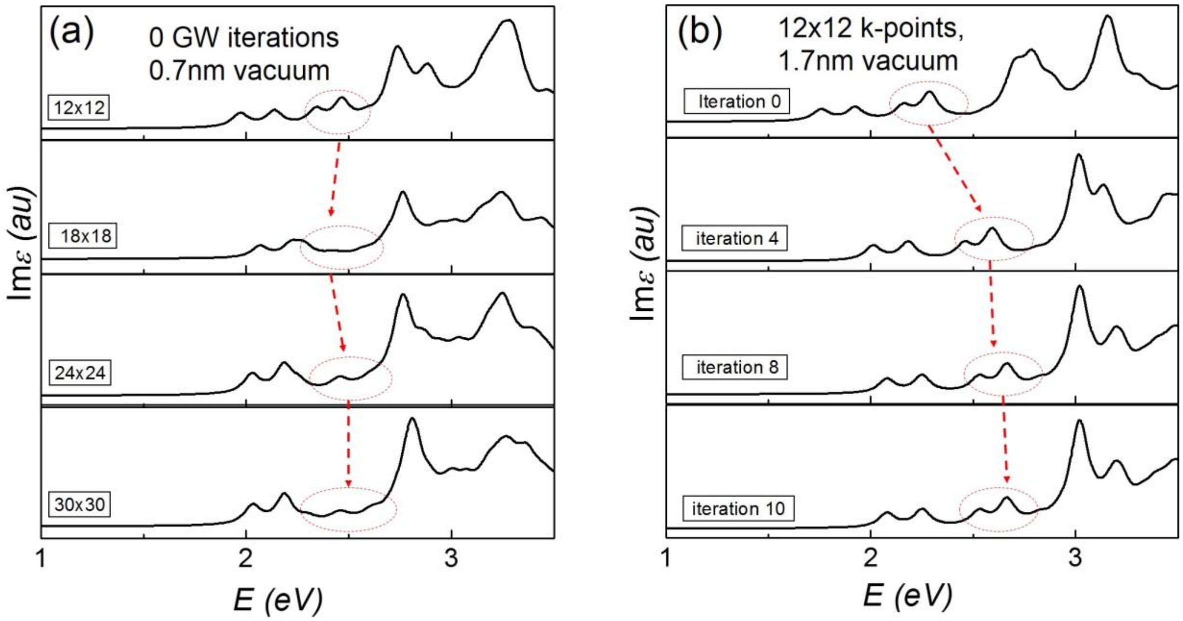

Figure S8: First-principles calculations for MoS_2 absorption spectra with different parameters. (a) Models of MoS_2 optical spectra with different k-point grids, no GW-iterations and small vacuum spacing. (b) Evolution of MoS_2 optical spectrum with iterations of the Green's function (G). These models are based on small k-point grid and large vacuum spacing. Red circles in (a) and (b) together with red arrows highlight the evolution of artificial features with k-point sampling/GW-iterations.

In order to choose optimal modeling parameters, we now discuss qualitatively how the factors discussed above affect the computational results.

i. *k-point sampling*: The k-point sampling $n \times n$ of a BZ allows us to map the particle's wavefunction to a real space area of only $n \times n$ unit cells. Thus, BZ with small sampling may cause artificial confinement of the exciton leading to artifacts in absorption spectrum (see below).

ii. *Vacuum layer thickness*: Using a periodic supercell in the calculation, modeling of 2-dimensional material is performed by its repetition in the *z*-direction with certain vacuum spacing. Too small vacuum layer results in increasing dielectric screening due to neighboring layers, which reduces the band gap and exciton binding energy.

iii. *Number of GW-iterations*: As GW-iterations are performed, we increase precision of particle's Green's function, which significantly affects particle's self-energy ($\Sigma = iGW$), the single-particle band structure and *e-h* interactions.

In order to perform accurate calculations within our computational resources, we have to optimize the most significant parameters. First, we calculate the optical spectrum using different k-points samplings as shown in (Fig.S8a). Since excitons in TMDCs are tightly bound, they are highly localized (within ~1 nm). A 12×12 grid corresponds to 12×12 unit cells in the real space, which corresponds to the confinement of excitons in lateral dimension of ~4 nm size. Thus, we expect that 12×12 k-point sampling will not significantly change the peak positions (Fig.S8a). We note that the features between ~2.3eV and ~2.7 eV (marked by red ovals) vary in amplitude and shape depending on the density of the mesh (Fig.S8a). We attribute these features to the artifacts of the calculation, which we discuss in details below.

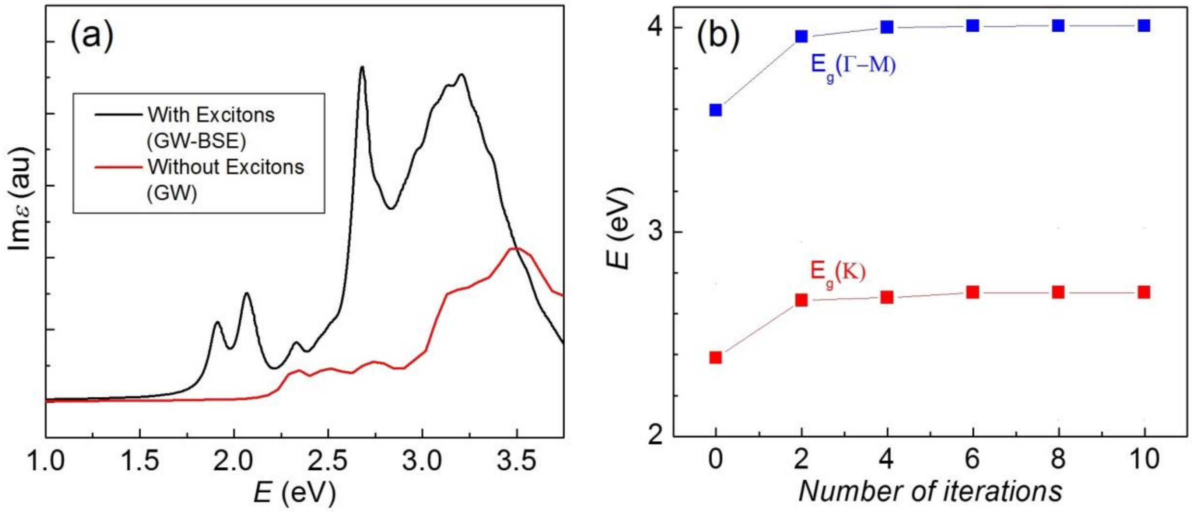

Figure S9: Optical spectra of MoS_2 with and without excitonic effects and their convergence.
(a) Optical spectra of MoS_2 with (black curve) and without (red curve) e-h interactions, performed for 24× 24 k-points, 0.7nm vacuum spacing and zero GW-iterations. We note that without e-h interactions, the absorption demonstrates a step-like increase at ~2.2eV (bandgap for this model is ~2.2 eV) as expected for hyperbolic bands at the K-valleys. Same features remain in place after inclusion of excitonic effects. (b) Evolution of the bandgap energies at different points of the BZ with GW_0 iterations. Red and blue squares indicate values of single-particle (GW_0) bandgap for K-point (location of A-exciton) and for the minimum between Γ- and M-points (one of the locations of C-exciton), respectively.

When photon energy exceeds the band gap, BZ sampling-related spatial confinement will cause discreet peaks instead of continuous absorption[27]. As we increase k-point sampling, peaks are expected to merge into continuum as evident from Fig.S8a. This is seen clearer in Fig.S9a, where we compare optical spectra calculated with and without excitonic effects using a (24×24) k-point sampling in a G_0W_0 calculation. The calculation without excitonic effects exhibits a plateau, starting at the band gap value ~2.2eV, and a residual peak at ~2.3 eV. These features remain in place after inclusion of the excitonic effects (via BSE), *i.e.*, these features are non-excitonic in nature. Hence, comparing spectra at different k-point sampling presented in Fig.S8a, we can distinguish between artificial ones (Fig.S8a, red dashed ovals) and real peaks.

Then, we increase vacuum layer to 17 Å and perform GW_0 calculations using a 12×12 k-point sampling (Fig.S8b) while tracking positions of real and artificial peaks. As evident from Fig.S8b and Fig.S9b, four iterations are necessary for the convergence of the band structure. We find that calculated A- and B-peak positions are higher than the experimental values by ~160 meV for MoS_2. Therefore, A- and B-peak positions alone cannot serve as a good criteria to compare the GW-BSE spectrum with the experimental results. However, we can compare the relative positions of A-, B- and C- peaks (E_C-E_A, E_C-E_B) instead of their absolute positions (E_A, E_B and E_C). The separation between these three peaks provides a basis for validating our calculations, *i.e.*, by comparing them with experimental data. To account for the mismatch between the measured and calculated peak positions (for example, ~160 meV for MoS_2), we manually red-shift the computational data to align calculated and experimental A- and B- peak positions (Fig. 2a, Fig. 3a).

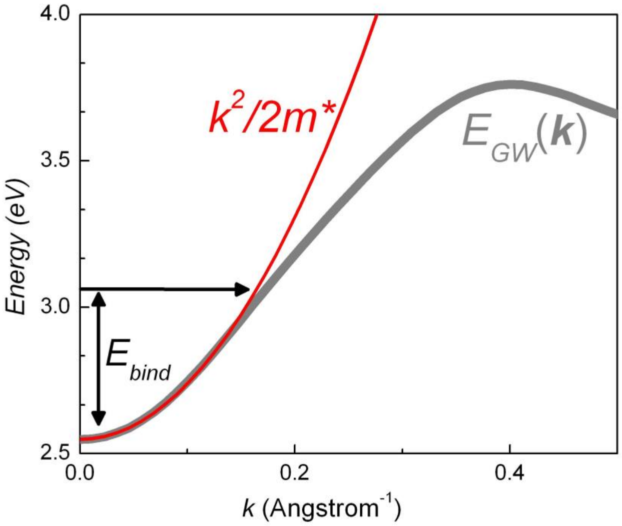

Figure S10: 2D-hydrogen model for an exciton in TMDC. We compare first-principles calculated optical bandstructure with the parabolic dispersion, expected in a 2D-hydrogen approximation. Here m is a reduced mass of an e-h pair. Vertical black arrow indicates the magnitude of the exciton binding energy.*

S7. 2D Hydrogen model for band edge excitons

To roughly estimate the binding energies and the oscillator strength of the ground and the excited states of excitons in TMDCs, we use a non-relativistic 2D hydrogen model. On the basis of our first-principle calculations, we estimate the reduced mass of an *e-h* pair m^*~$0.2m_e$ (here m_e is the electron mass). If we assume the dielectric constant of MoS_2 to be ~4.2[28], the binding energy of an A-exciton, according to the 2D-hydrogen model with *1/r* potenital, can be estimated as[29]

$$E_{bind} = \frac{1}{2(n-1/2)^2}\frac{m^* e^4}{(4\pi\varepsilon)^2\hbar^2},$$

giving the ground state binding energy E_{bind}~620 meV. This value agrees well with the experimentally estimated value ~570 meV. In the same model, the oscillator strength decreases[30] with n as $f_n \propto$

$1/(n\text{-}1/2)^3$. For example, the exciton's first excited state has oscillator strength smaller by a factor f_1 / f_2 = 27 than that for the ground state.

Finally, we discuss the validity of the 2D hydrogen approximation. In order to study the physics of excitons, it is convenient to work in the center-of-mass (CM) frame. In this frame of reference, the dispersion of particles constituting an exciton is given by the difference between the valence and the conduction bands[31] (*i.e.*, optical band structure). On the other hand, in a non-relativistic 2D-hydrogen model, the dispersion relation is parabolic. Thus, the non-relativistic 2D-hydrogen model is considered a good approximation if the calculated optical band structure is close to the parabolic dispersion at the exciton binding energies. Fig.S10 shows a calculated optical bandstructure (grey curve) of MoS_2 near the K- point, and its parabolic approximation based on the 2D-hydrogen model (red curve). At relevant energies (E_g+E_{bind}, marked by horizontal arrow), we see ~10% deviation of the parabolic approximation from the calculated GW dispersion. This implies that 2D-hydroden model is sufficient for qualitative treatment of the A/B-excitons.

References:

1 Lee, C. *et al.* Anomalous Lattice Vibrations of Single- and Few-Layer MoS_2. *ACS Nano* **4**, 2695-2700 (2010).

2 Dean, C. R. *et al.* Boron nitride substrates for high-quality graphene electronics. *Nat Nanotechnol* **5**, 722-726 (2010).

3 Bolotin, K. I. *et al.* Ultrahigh electron mobility in suspended graphene. *Solid State Communications* **146**, 351-355 (2008).

4 Mak, K. F., Lee, C., Hone, J., Shan, J. & Heinz, T. F. Atomically Thin MoS_2: A New Direct-Gap Semiconductor. *Phys Rev Lett* **105**, 136805 (2010).

5 Conley, H. J. *et al.* Bandgap Engineering of Strained Monolayer and Bilayer MoS_2. *Nano Lett* **13**, 3626-3630 (2013).

6 Chakraborty, B. *et al.* Symmetry-dependent phonon renormalization in monolayer MoS_2 transistor. *Physical Review B* **85**, 161403 (2012).

7 Freitag, M., Low, T., Xia, F. N. & Avouris, P. Photoconductivity of biased graphene. *Nat Photonics* **7**, 53-59 (2013).

8 Mohite, A. D., Gopinath, P., Shah, H. M. & Alphenaar, B. W. Exciton dissociation and stark effect in the carbon nanotube photocurrent spectrum. *Nano Lett* **8**, 142-146 (2008).

9 Tongay, S. *et al.* Defects activated photoluminescence in two-dimensional semiconductors: interplay between bound, charged, and free excitons. *Sci. Rep.* **3**, 2657 (2013).

10 Eda, G. *et al.* Photoluminescence from Chemically Exfoliated MoS_2 *Nano Lett* **12**, 526-526 (2012).

11 Nagpal, P. & Klimov, V. I. Role of mid-gap states in charge transport and photoconductivity in semiconductor nanocrystal films. *Nat Commun* **2** (2011).

12 Schroder, D. K. *Semiconductor material and device characterization*. 3rd edn, (Wiley, 2006).

13 Kam, K. K. & Parkinson, B. A. Detailed Photocurrent Spectroscopy of the Semiconducting Group-Vi Transition-Metal Dichalcogenides. *J Phys Chem-Us* **86**, 463-467 (1982).

14 Korn, T., Heydrich, S., Hirmer, M., Schmutzler, J. & Schüller, C. Low-temperature photocarrier dynamics in monolayer MoS2. *Appl Phys Lett* **99**, 102109 (2011).

15 Zeng, H. L., Dai, J. F., Yao, W., Xiao, D. & Cui, X. D. Valley polarization in MoS_2 monolayers by optical pumping. *Nat Nanotechnol* **7**, 490-493 (2012).

16 Dolui, K., Rungger, I. & Sanvito, S. Origin of the n-type and p-type conductivity of MoS_2 monolayers on a SiO_2 substrate. *Physical Review B* **87**, 165402 (2013).

17 Kresse, G. & Furthmüller, J. Efficient iterative schemes for ab initio total-energy calculations using a plane-wave basis set. *Physical Review B* **54**, 11169-11186 (1996).

18 Perdew, J. P., Burke, K. & Ernzerhof, M. Generalized Gradient Approximation Made Simple. *Phys Rev Lett* **77**, 3865-3868 (1996).

19 Blöchl, P. E. Projector augmented-wave method. *Physical Review B* **50**, 17953-17979 (1994).

20 Kresse, G. & Joubert, D. From ultrasoft pseudopotentials to the projector augmented-wave method. *Physical Review B* **59**, 1758-1775 (1999).

21 Grimme, S. Semiempirical GGA-type density functional constructed with a long-range dispersion correction. *Journal of Computational Chemistry* **27**, 1787-1799 (2006).

22 Shishkin, M. & Kresse, G. Implementation and performance of the frequency-dependent GW method within the PAW framework. *Physical Review B* **74**, 035101 (2006).

23 Mostofi, A. A. *et al.* wannier90: A tool for obtaining maximally-localised Wannier functions. *Computer Physics Communications* **178**, 685-699 (2008).

24 Rohlfing, M. & Louie, S. G. Electron-Hole Excitations in Semiconductors and Insulators. *Phys Rev Lett* **81**, 2312-2315 (1998).

25 Albrecht, S., Reining, L., Del Sole, R. & Onida, G. Excitonic Effects in the Optical Properties. *physica status solidi (a)* **170**, 189-197 (1998).

26 Onida, G., Reining, L. & Rubio, A. Electronic excitations: density-functional versus many-body Green's-function approaches. *Reviews of Modern Physics* **74**, 601-659 (2002).

27 Molina-Sánchez, A., Sangalli, D., Hummer, K., Marini, A. & Wirtz, L. Effect of spin-orbit interaction on the optical spectra of single-layer, double-layer, and bulk MoS_2. *Physical Review B* **88**, 045412 (2013).

28 Cheiwchanchamnangij, T. & Lambrecht, W. R. L. Quasiparticle band structure calculation of monolayer, bilayer, and bulk MoS2. *Physical Review B* **85**, doi:205302 (2012).

29 Yang, X. L., Guo, S. H., Chan, F. T., Wong, K. W. & Ching, W. Y. Analytic solution of a two-dimensional hydrogen atom. I. Nonrelativistic theory. *Physical Review A* **43**, 1186-1196 (1991).

30 Yu, P. Y. & Cardona, M. *Fundamentals of semiconductors : physics and materials properties*. 2nd updated edn, (Springer, 1999).

31 Dresselhaus, G. Effective mass approximation for excitons. *Journal of Physics and Chemistry of Solids* **1**, 14-22 (1956).
